# Locating critical events in AFM force measurements by means of one-dimensional convolutional neural networks

**DOI:** 10.1038/s41598-022-17124-z

**Published:** 2022-07-29

**Authors:** Javier Sotres, Hannah Boyd, Juan F. Gonzalez-Martinez

**Affiliations:** 1 Department of Biomedical Science, Faculty of Health and Society, Malmö University, 20506 Malmö, Sweden; 2 Biofilms-Research Center for Biointerfaces, Malmö University, 20506 Malmö, Sweden; 3 Department of Applied Physics, Technical University of Cartagena, 30202 Cartagena, Spain

**Keywords:** Materials science, Nanoscience and technology, Physics

## Abstract

Atomic Force Microscopy (AFM) force measurements are a powerful tool for the nano-scale characterization of surface properties. However, the analysis of force measurements requires several processing steps. One is locating different type of events e.g., contact point, adhesions and indentations. At present, there is a lack of algorithms that can automate this process in a reliable way for different types of samples. Moreover, because of their stochastic nature, the acquisition and analysis of a high number of force measurements is typically required. This can result in these experiments becoming an overwhelming task if their analysis is not automated. Here, we propose a Machine Learning approach, the use of one-dimensional convolutional neural networks, to locate specific events within AFM force measurements. Specifically, we focus on locating the contact point, a critical step for the accurate quantification of mechanical properties as well as long-range interactions. We validate this approach on force measurements obtained both on hard and soft surfaces. This approach, which could be easily used to also locate other events e.g., indentations and adhesions, has the potential to significantly facilitate and automate the analysis of AFM force measurements and, therefore, the use of this technique by a wider community.

## Introduction

Since its invention^1^, the Atomic Force Microscope (AFM) has not only been revealed as a powerful tool to visualize surfaces at the nanoscale, but also to characterize different sample properties. The most common procedure for these characterizations is based on the acquisition of the so-called normal force curves, a method also known as force spectroscopy, where the force between the AFM probe and the investigated sample is registered at different positions along the sample normal direction^2^. AFM force curves have been widely used to perform a vast variety of e.g., chemical^3,4^, mechanical^5–7^ and electrical^8,9^ characterizations.

The analysis of force curves requires a number of processing steps, often with the goal to transform the experimental raw data i.e., position sensitive photodetector (PSD) values vs sample vertical positions, into a representation of forces vs probe-sample separations. This then allows, for example, the identification of the forces and separations at which certain events like adhesions and indentations occur, or to fit different sections of the curves to physical laws in order to quantify sample properties. While it is fairly easy to automate some of these required processing steps, for some others the input of AFM experienced users is often required. Moreover, because of the stochastic nature of force measurements and the lateral heterogeneity of most samples, the acquisition and analysis of a high number of force curves is typically required to obtain statistically significant results. This can result in the force spectroscopy experiments becoming an overwhelming task if their analysis are not automatized.

Here, we explored a Machine Learning (ML) approach for one of the most common processing steps required for AFM force curves analysis: the precise location of events of interest. As mentioned, this task can include locating indentation and adhesion events. In this work, we focused instead on locating the contact point. Accurately locating the contact point or point of zero distance is critical for representing a force curve in terms of force vs probe-sample separation. This then allows, for example, to correctly define indentation distances that in turn allow the characterization of mechanical properties of the samples^10^. Correctly determining probe-sample distances is also critical for the analysis of long-range interactions^11^. In the case of hard surfaces this is relatively simple. The contact point is defined as the point where two regions of the curve, which are characterized by clearly differentiated slopes, intersect each other. This can be further facilitated in the case that a jump-to-contact event is observed. However, locating the contact point is significantly more difficult in the case of soft surfaces, and even more if they exhibit long-range interactions. In this case, the slope change between the contact and the non-contact regions might not be clearly differentiated and jump-to-contact events are not always observed. Typically, an accurate location of the contact point in these cases requires the attention of an experienced AFM user.

Different approaches have been proposed for the automated detection of the contact point in AFM force curves. In the literature there are two main trends. One focuses on determining the point for which the deflection of the cantilever increases above a threshold value set by the user, typically a multiple of the signal to noise ratio of the non-contact region of the force curve^12,13^. This approach can be complemented with fitting the contact region to different models that include the contact point as a fitting parameter, allowing the contact point to be re-defined in a second step^14^. This strategy has also been combined with additional algorithms to separate the non-linear and linear regions of the contact regime^13^. The second approach focuses on determining the point for which the first^15^ or second^16^ derivative of the force curve surpass a pre-defined threshold. Additionally, this approach is usually preceded by the prior application of smoothing filters. While powerful and well-tested, these approaches require the values of different parameters to be pre-defined e.g., window size for calculating the signal-to-noise ratio or the curve derivatives, threshold values and ratios of the total curve length corresponding to the non-contact region. However, the parameter values that allow to accurately locate the point of interest depend on the curve shape. This will in turn depend on specifics of the investigated sample e.g., mechanical properties, presence of indentation events and long-range interactions, as well as on several acquisition parameters e.g., ramp length of the non-contact region, maximum applied load, sampling frequency and number of averages per sample vertical position. Here, we explore a different approach to this problem: the use of ML algorithms, specifically one-dimensional (1D) convolutional neural networks (CNNs). Based on how neural networks excel in identifying patterns in heterogeneous data samples, we hypothesized that they would show better generalization i.e., an ability to accurately locate the contact point without the need to search for optimal values for parameters of the event location algorithm for each set of curves obtained under different environmental or acquisition conditions.

ML techniques have already started to be applied in the field of AFM, even thought to a lower extent than in other microscopies. This includes works on the automation of AFM operation^17–21^ but mostly on AFM data analysis, both in the context of image analysis^22–29^ and force curves analysis^30–32^. However, in the context of force curves analysis the goal of ML techniques has mostly been to classify the curves e.g., according to the type of adhesion exhibited. Here, we were interested in inference instead, specifically in using these techniques to locate the position of critical events, with a specific focus on the contact point. As mentioned, for this goal we propose the use of 1D CNNs. Convolutional neural networks were developed^33^ with the main goal of classifying two-dimensional (2D) data i.e., images. Since then, they have shown an extraordinary performance in this field^34^. Moreover, 2D CNNs have also been used for inference purposes. An example of relevance for this work being the location of specific objects within images^35–38^. Prompted by the success of 2D CNNs, 1D CNNs were developed for the context of analysis of 1D signals and rapidly achieved a high performance in a diversity of applications^39^. Here, we explored the performance of two different 1D CNNs architectures to locate the contact point in force measurements performed in two different systems: (i) a hard surface in the presence of long-range forces (mica in aqueous solutions) and (ii) a soft surface in the presence of long-range forces (salivary pellicles in aqueous solutions).

## Results and discussion

**Figure 1:**
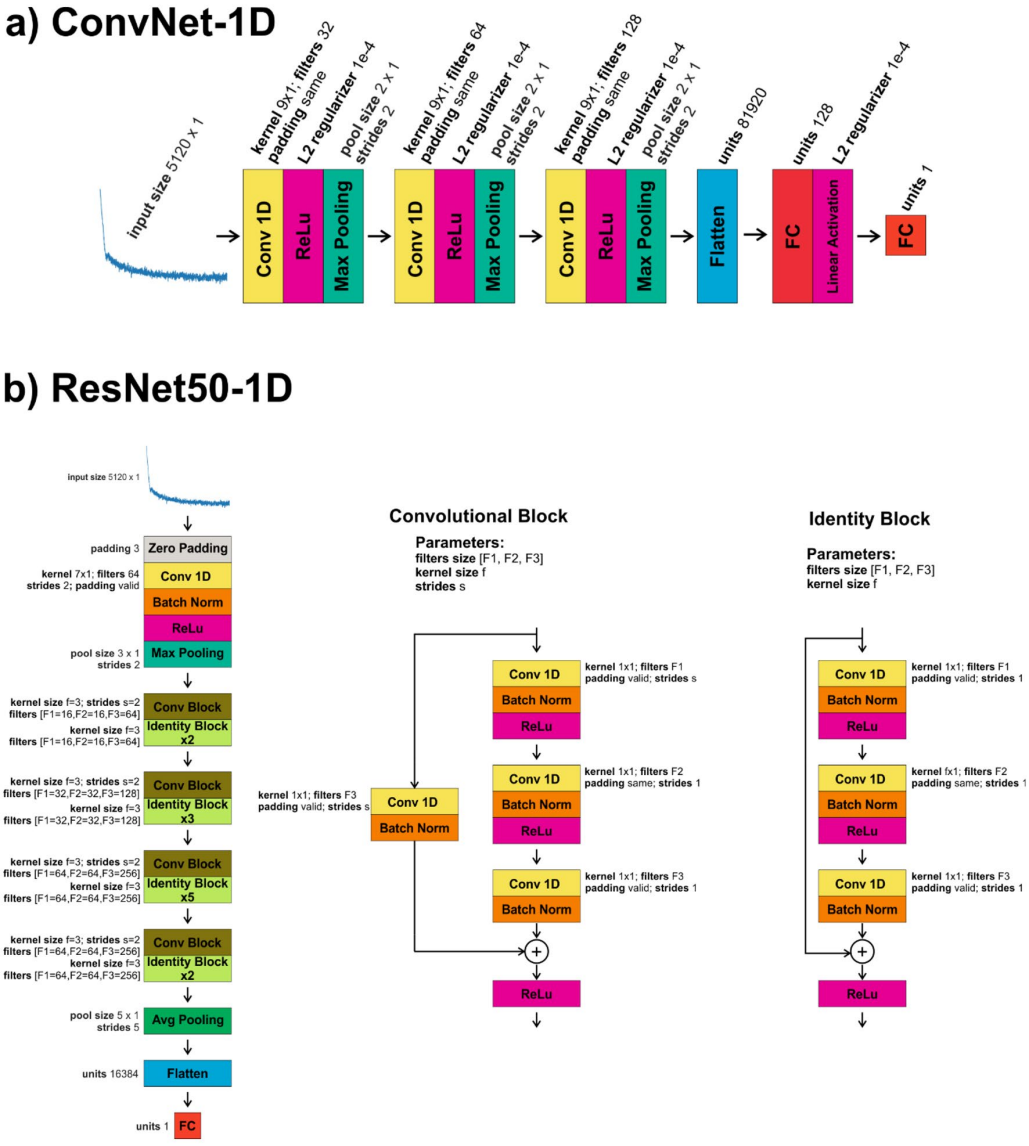
Convolutional neural networks used in this study: (**a**) ConvNet-1D and (**b**) ResNet-1D.

Since the development of convolutional neural networks, one of the most common basic architectures consisted of combining blocks containing convolutional layers followed by sub-sampling layers in the beginning of the network, these blocks then are followed by fully connected layers^33,40^. This type of architecture has also been proved successful in the analysis of 1D data just by using one-dimension convolutional layers rather than two-dimensional ones^41–44^. Subsequently, we also explored this architecture. Specifically, we report results obtained using the architecture depicted in Fig. 1a, from now on named ConvNet-1D. It consists in three blocks of 1D convolutional layers and sub-sampling (max-pooling) layers, followed by two fully connected layers (specific layers parameters are provided in Fig. 1a), the last one consisting of only one unit accounting for the predicted position of the critical event to be located, in this case the contact point within the experimental force curves.

**Figure 2:**
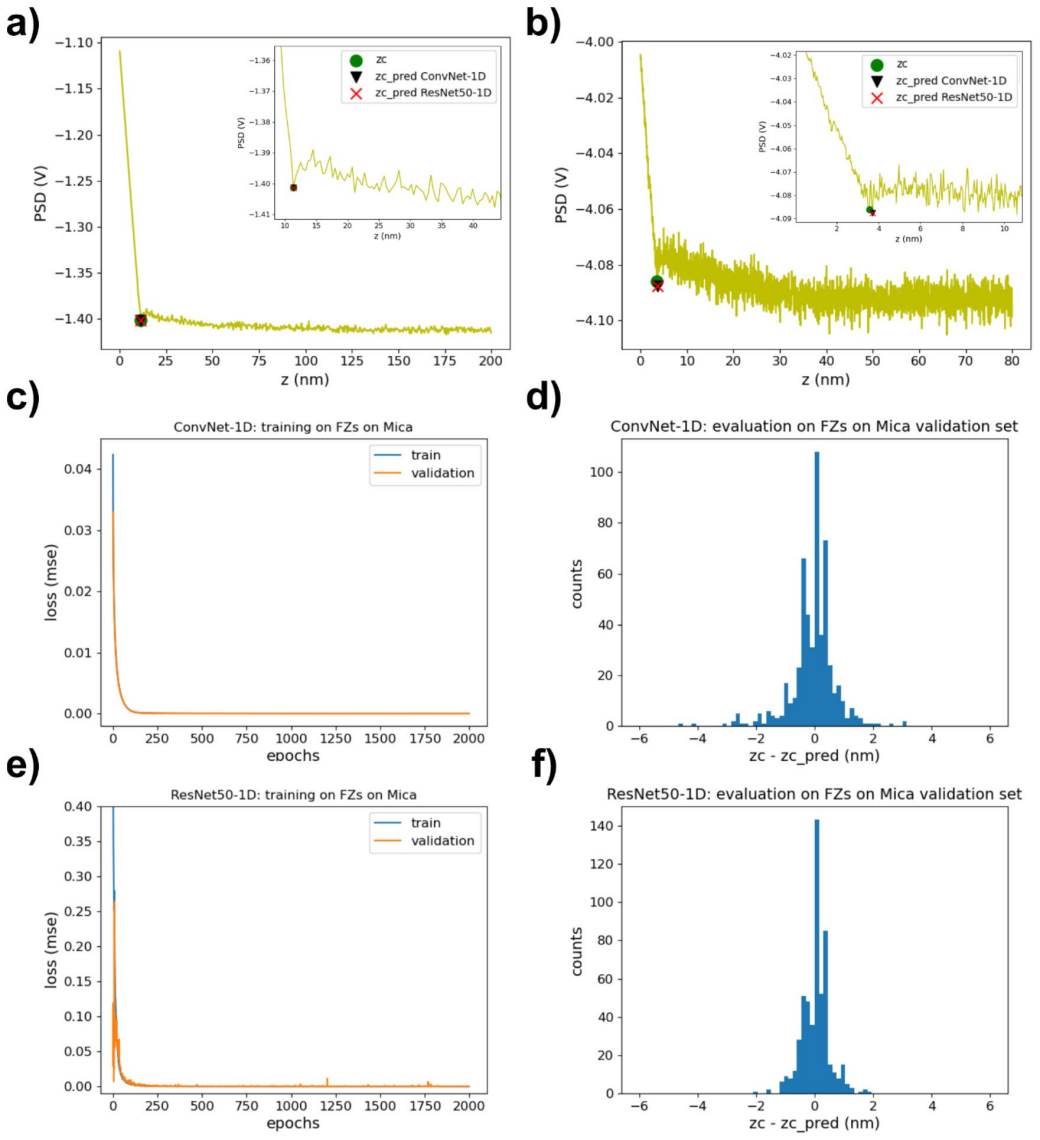
**(a**) and (**b**) Representative examples of force curves obtained with Si_3_N_4_ AFM tips on mica surfaces in NaCl aqueous solutions. The insets correspond to zooms of the contact point region. The curves also contain markers for the labelled contact point (green circle) as well as the contact points predicted by ConvNet-1D (black triangle) and by ResNet50-1D (red cross). (**c**) Evolution of the mse loss function i.e., the mean squared differences between labelled and ConvNet-1D predicted normalized sample positions corresponding to the contact point, obtained while training ConvNet-1D on force curves on mica. Loss values for both the training and validation sets are shown. Total training time = 1096 s (~ 0.55 s/epoch). (**d**) Distribution of the difference between the sample positions of the hand-labelled, zc, and the ConvNet-1D predicted, zc_pred, contact points (recalculated for the non-normalized force curves). (**e**) Loss (mse) values for the same training and validation sets obtained in this case while training the ResNet50-1D network. Total training time = 4366 s (~ 2.18 s/epoch). (**f**) zc-zc_pred distribution for the force curves on mica validation set as predicted by ResNet50-1D.

We first investigated the performance of ConvNet-1D to locate the contact point in force curves performed with Si_3_N_4_ tips on mica in aqueous solutions. Specifically, we experimentally collected a set of 1806 force curves obtained in 100 mM, 10 mM and 1 mM NaCl solutions using three different probe/sample pairs for each type of solution. Representative examples are shown in Fig. 2a,b. These force curves exhibit long range electrostatic forces when interacting at neutral pH values, as a result of both sample and tip being negatively charged^8^. At very short separations, the attractive van der Waals forces overcome the repulsive electrostatic force, often resulting in a mechanical instability known as jump-to–contact^2^. From this point, mechanical contact is established and the cantilever deflection increases approximately with the same rate as the sample displacement considering that mica surfaces (effective stiffness as measured by AFM ~ 82 N m^−1^)^45^ are significantly harder than the employed cantilevers (nominal force constant 0.1 N m^−1^). First, we manually labelled the contact point in all collected force curves. Then, we randomly divided them into a training (70% of the curves) and a validation (30% of the curves) data sets. The curves shown in Fig. 2a,b correspond to the validation data set. Neural networks require a fixed input data size, and force curves are usually collected at a variety of sizes/points. This was also the case for those used in this work. To account for this, all curves were interpolated up to a total size of 5120 points, a value higher than the size of any of the collected experimental curves. Additionally, before feeding the curves to the networks, they were normalized both in the vertical (PSD values) and horizontal (sample displacement) axis. The output of the network will then be the normalized sample position of the contact point. Then, we trained the ConvNet-1D network on the training set using the mean square error (mse) loss function. The evolution of the mse loss while training the network over 2000 epochs is shown in Fig. 2c for both the training and validation data sets. No signs of exploding gradients, vanishing gradients or overfitting were observed. However, for avoiding overfitting we needed to incorporate L2 regularization in the different layers of the network. We then chose the weights obtained for the epoch with the minimum loss. As a quantity to validate the performance of the network, we used the difference between the sample positions of the hand-labelled, zc, and the predicted, zc_pred, contact points (recalculated for the non-normalized force curves). The distribution of this quantity for the validation data set is shown in Fig. 2d. The mean and standard deviation values for this distribution were 0.06 ± 0.78 nm (Table 1). Overall, we can see how a fairly simple and shallow architecture as that of ConvNet-1D provided excellent accuracy.

**Table 1:** Mean and standard deviation values for the difference between the sample positions of the labelled and predicted (by the two networks used in this work) contact points for all validation and test data sets.

	zc-zc_pred (nm)	
	ConvNet-1D	ResNet50-1D
Mica—validation set (NaCl 100 mM/10 mM/1 mM)	0.06 ± 0.78	0.03 ± 0.46
Pellicle on Mica—validation set (NaCl 100 mM/10 mM)	0.04 ± 1.19	0.01 ± 0.71
Pellicle on Mica—test set (NaCl 1 mM)	1.20 ± 1.80	0.20 ± 0.97

**Figure 3:**
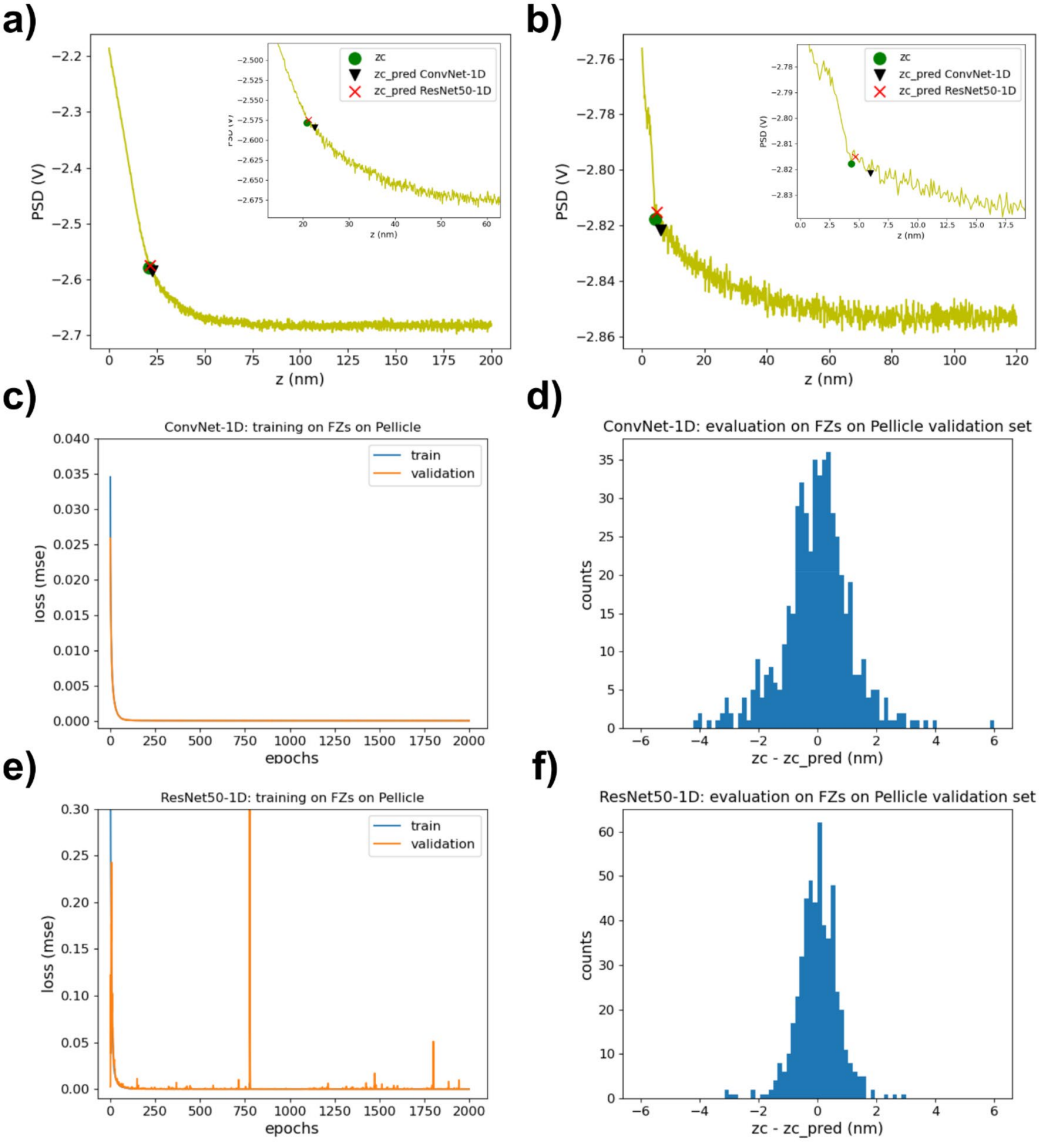
(**a**) and (**b**) Representative examples of force curves obtained with Si_3_N_4_ AFM tips on salivary pellicles in NaCl (100 mM and 10 mM) aqueous solutions. The insets correspond to zooms of the contact point region. The curves also contain markers for the labelled contact point (green circle) as well as the contact points predicted by ConvNet-1D (black triangle) and by ResNet50-1D (red cross). (**c**) Evolution of the loss mse function while training ConvNet-1D on force curves on pellicles. Loss values for both the training and validation sets are shown. Total training time = 1615 s (~ 0.81 s/epoch). (**d**) Distribution of the difference between the sample positions of the hand-labelled, zc, and the ConvNet-1D predicted, zc_pred, contact points (recalculated for the non-normalized force curves). (**e**) Loss (mse) values for the same training and validation sets obtained while training the ResNet50-1D network on the same data set. Total training time = 6760 s (~ 3.38 s/epoch). (**f**) zc-zc_pred distribution for the force curves on the pellicles validation set using the values predicted by ResNet50-1D.

We then investigated the performance of ConvNet-1D for locating the contact point on force curves performed on salivary pellicles with Si_3_N_4_ tips in aqueous NaCl solutions. Salivary pellicles are nm-thick proteinaceous films that rapidly form on almost any surface when exposed to saliva. They do not only confer oral surfaces with extraordinary protection against chemical and mechanical insults^46,47^. Salivary pellicles also constitute excellent aqueous boundary lubricants with the ability to provide low friction coefficients and high yield strengths to a wide variety of tribological contacts^48–51^. Several experimental results point towards a two-layer model for salivary pellicles reconstituted at solid–liquid interfaces, with an inner thin dense layer, mainly formed by low molecular weight proteins, and an outer thick diffuse layer mainly formed by the long glycoproteins known as mucins^46^. AFM force measurements are one of the main techniques that contributed to elucidate the structure of salivary pellicles^11,52^. AFM force measurements have also been used to determine how this structure is affected by oral health products like surfactants^53^. Force curves performed on salivary pellicles, for which representative examples are shown in Fig. 3a,b, resemble up to some extent those obtained on mica surfaces (Fig. 2a,b). When approaching probe and sample, a long range interaction with an exponential-like dependence with distance is initially observed. This is originated by the repulsive steric interaction that develops between the AFM probe and the diffuse pellicle outer layer. At shorter separations, a transition to a different regime is observed where the cantilever deflection exhibits a steeper dependence with the separation from the probe. This regime is typically well-fitted by mechanical contact models. In this system, the contact point delimits the transition between these two different regimes, and its accurate determination is critical for a correct interpretation of the mechanical properties of the pellicle and, therefore, of its structure. However, the position of the contact point in this system is not as clear as in the case of force curves performed on mica. Often, the slope difference between the steric and the mechanical contact regime is not clearly differentiated because of the sample softness. The jump-to-contact event is not as prominent either, or even sometimes absent. The location of the contact point can be facilitated though by the fact that the deflection noise typically decreases when entering the contact region^54^. Overall, the location of the contact point in this case is fairly difficult to determine by means of hard coded algorithms, and typically requires that an expert user analyzes each of the registered force curves.

We initially tested the performance of ConvNet-1D for locating the contact point on a set of force curves on salivary pellicles obtained both in NaCl 100 mM and NaCl 10 mM aqueous solutions, using three different probe/sample pairs for each type of solution. Specifically, we experimentally collected a total of 2584 force curves for this system. We then manually labelled the contact point in all curves and randomly divided them into a training (80% of the curves) and a validation (20% of the curves) sets (the ratio of the curves used for validation was lower than that used for the dataset of curves performed on mica surfaces as the total number of curves was higher in the current case). The curves in Fig. 3a,b correspond to the validation set. Before feeding the curves to the network, they were interpolated and normalized as previously described. The network was trained again over 2000 epochs. The mse loss for both the train and validation data sets during training is shown in Fig. 3c. As in the case of force curves obtained on mica, no signs of exploding gradients, vanishing gradients or overfitting were observed. Here, we also chose for prediction purposes the weights obtained for the epoch with the minimum validation loss. The distribution of the difference between the sample positions of the labelled and predicted contact points (recalculated for the non-normalized force curves) is presented in Fig. 3d. The mean and standard deviation values for this distribution were 0.04 ± 1.19 nm (Table 1). Overall, our results show again a good performance of ConvNet-1D, even though the distribution of values was slightly wider than that obtained when applying the same network to locate contact points in force curves performed on mica. Of relevance is that ConvNet-1D was able to easily distinguish contact points from indentations, as observed in Fig. 3b.

**Figure 4:**
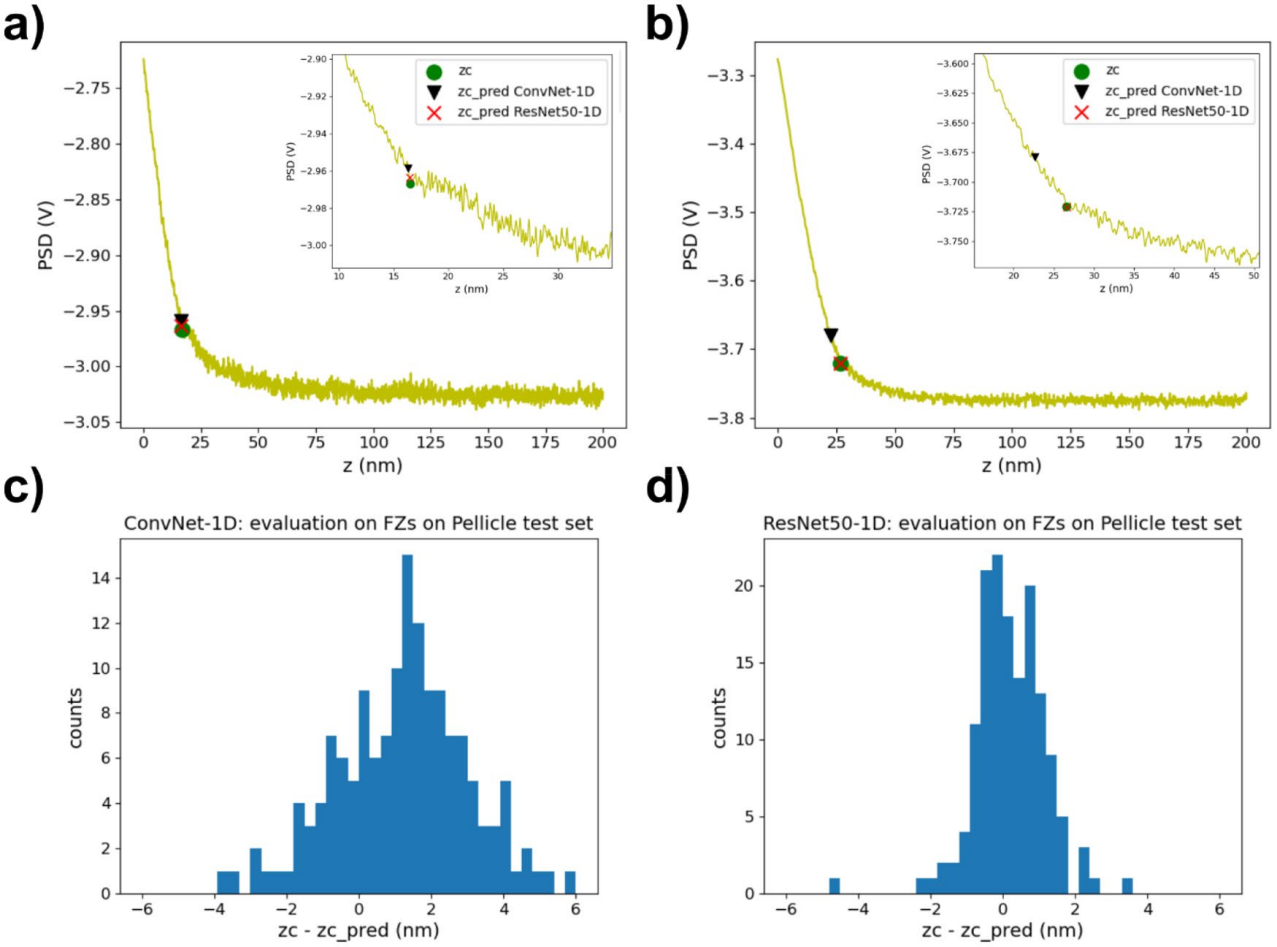
(**a**) and (**b**) Representative examples from the test set of force curves obtained with Si_3_N_4_ AFM tips on salivary pellicles in NaCl 1 mM aqueous solutions. The insets correspond to zooms of the contact point region. The curves also contain markers for the labelled contact point (green circle) as well as the contact points predicted by ConvNet-1D (black triangle) and by ResNet50-1D (red cross). (**c**) and (**d**) zc-zc_pred distributions for this test data set, obtained with the ConvNet-1D and ResNet50-1D networks respectively. The total inference time when using ConvNet-1D was 1.3 s (~ 8.5 ms/curve) whereas for ResNet50-1D it was 2.3 ms (~ 15 ms/curve).

Subsequently, we investigated the generalization of ConvNet-1D for locating contact points in a set of curves not used for training. For this, we tested the already trained network on a set of 153 force curves corresponding to a different salivary pellicle sample/Si_3_N_4_ AFM tip pair than those used for training the network. This test set was also obtained in different conditions i.e., NaCl 1 mM. Representative curves are shown in Fig. 4a,b. The distribution of the difference between the sample positions of the labelled, zc, and ConvNet-1D predicted, zc_pred, contact points (recalculated for the non-normalized force curves) is presented in Fig. 4c. The mean and standard deviation values for this distribution were 1.20 ± 1.80 nm (Table 1). While still reasonable, it is clear that the performance of ConvNet-1D decreased when applied to data different than that used for training.

We then explored whether we could improve our results by means of a different network architecture. First, we explored different hyper-parameter values for ConvNet-1D, but we did not find a set of values that significantly improved its performance. Small increments in the network depth did not have a significant effect either. We then investigated the use of significantly deeper networks. Specifically, we focused on residual networks, which contain residual blocks that include short paths (Fig. 1b) with the ability to carry gradients throughout very deep networks mitigating in this way the problem of vanishing gradients^55^. We used as a starting point the ResNet-50 network^56^ as implemented in Keras (^57^, https://t.ly/uLupD) and modified it in order to use 1D rather than 2D convolutional layers. The specific implemented architecture, from now on named ResNet50-1D, is shown in Fig. 1b. The train and validation mse losses obtained while training ResNet50-1D on force curves on mica and on salivary pellicles are shown in Figs. 2e and 3e respectively. The distribution of the difference between the sample positions of the hand-labelled and predicted contact points (recalculated for the non-normalized force curves) is presented in Figs. 2f and 3f for the validation sets of force curves performed on mica and salivary pellicles respectively. The mean and standard deviation values for these distributions were 0.03 ± 0.46 nm for the mica validation set and 0.01 ± 0.71 nm for the pellicle validation set (Table 1). Therefore, already on these sets ResNet50-1D exhibited a slightly better performance than ConvNet-1D, with narrower zc-zc_pred distributions. We subsequently tested the performance of the already trained ResNet50-1D network on the test set consisting of force curves on salivary pellicles in NaCl 1 mM not used for training the network. The corresponding zc-zc_pred distribution is shown in Fig. 4d. The mean and standard deviation values for this distribution was 0.20 ± 0.97 nm (Table 1) i.e., the width of the distribution was approximately half than that obtained with ConvNet-1D.

Overall, our results prove that 1D CNNs can be used for accurately locating the contact point in AFM force curves. This was quantified by comparing the hand-labelled and the predicted contact point positions, zc-zc_pred, which was below 1 nm in most cases (Table 1). Common 1D CNNs networks with a relative simple and shallow architecture, like ConvNet-1D, can already accomplish this task with good accuracy. However, we also showed that deeper networks that make use of residual blocks, like ResNet50-1D, result in a more accurate location of this event as well as better generalization i.e., performance when used on data from a different set than that used for training. Obviously, this improved performance comes at a cost in terms of time. As reported, the time needed for training the networks was a factor ~ 4 higher for ResNet50-1D (~ 2.18 s/epoch on the mica train & validation datasets; ~ 3.38 s/epoch on the pellicle train & validation datasets) than for ConvNet-1D (~ 0.55 s/epoch on the mica training & validation datasets; ~ 0.81 s/epoch on the pellicle training & validation datasets). Nevertheless, this increase in training time should not be a major limitation for choosing deeper networks (as seen in Figs. 2 and 3, for all datasets the losses for both models converged to an almost constant value after ~ 250 epochs) unless one is dealing with very large datasets or only has access to low speed processors for training the networks. Inference time should also not be a major limitation for choosing ResNet50-1D like architectures (~ 15 ms/curve) over ConvNet-1D like ones (~ 8.5 ms/curve). It is worth highlighting the improvement this represents in comparison to a hand-labeling approach, where the user would spend in the best of cases few seconds on each curve.

The inference time is similar though to that achieved by other event locating algorithms. For instance, Offroy and co-workers^13^ reported (~ 23 ms/curve) for their algorithm based on an increase of the cantilever deflection above the value of the signal-to-noise ratio of the non-contact region of the curve, or even probably slightly higher taking into account that they used a standard PC and that their algorithm also calculated the border between the linear and non-linear regimes of the contact region. However, it is also worth highlighting the generalization capability of the proposed ML-based approach. As stated in the Methods section, the datasets used in this work contained force curves obtained while randomly varying acquisition parameters like the ramp length, the maximum applied load, the sampling frequency and the ramp velocity. This heterogeneity is exemplified by the differences exhibited between the curves plotted in the figures of the manuscript for each dataset. Moreover, our datasets combined curves obtained in different environments (ionic strength), which result in different long-range interactions. Interestingly, our results show that 1D CNNs could accurately locate the contact point despite these differences, even in the presence of unexpected events like indentations (Fig. 3b). In contrast with previous proposed approaches, this could be achieved without pre-defining values for parameters of the event location algorithm.

The specific focus of this work was on locating the contact point. However, we see no limitations in using the same approach to locate other events in AFM force curves e.g., indentations and adhesions. It should also be possible to use architectures that would allow locating a variable number of different type of events within the same AFM curve by using similar approaches as the available object detector networks for 2D data. Overall, we foresee that these ML-based approaches will significantly facilitate AFM data analysis and, therefore, the use of this technique by a wider community.

## Methods

### Materials

All water used to prepare salt solutions was of ultrahigh quality (UHQ), processed in an Elgastat UHQ II apparatus (Elga Ltd., High Wycombe, Bucks, England). Mica sheets were purchased from Electron Microscopy Sciences (Hatfield, PA).

For saliva experiments, unstimulated human whole saliva (HWS) was collected from two healthy donors, after they provided written informed consent, using the protocol described in^58^, pooled and then used immediately. Ethical approval was obtained from the Swedish Ethical Review Authority through the committee of research ethics at Lund University (2018/42), and saliva collection was performed following the protocol in the mentioned ethical permit, which was in accordance with the Declaration of Helsinki.

Unless otherwise specified, all other chemicals used were of at least analytical grade.

### Force spectroscopy

A commercial Atomic Force Microscope (AFM) setup equipped with a liquid cell (MultiMode 8 SPM with a NanoScope V control unit, Bruker AXS, Santa Barbara CA) was employed for the acquisition of force curves. Rectangular silicon nitride levers with a nominal normal spring constant of 0.1 N m^−1^ were employed in all the experiments (OMLC-RC800PSA, Olympus, Japan). The choice of cantilevers with this spring constant was based on our previous experience, as they exhibit significant deflection values that allow investigating electrostatic forces on mica^8^ as well as steric forces and deformation of salivary pellicles^11,53^. Choosing cantilevers with significantly higher spring constants would have resulted in a negligible influence of long-range forces or mechanical deformation on the measured cantilever deflection, in which case the location of the contact point becomes a straightforward problem to solve. Before every experiment, tips were rubbed against a clean freshly cleaved mica surface, a procedure that also leads to the removal of asperities and achieves a smooth tip surface^59^.

Force spectroscopy on mica experiments were performed on freshly cleaved mica sheets. For force spectroscopy on salivary pellicles, freshly cleaved mica sheets (~ 1 cm^2^) were drop-coated with 100 µL of HWS, and subsequently rinsed with NaCl 100 mM after 1 h. Samples were then immediately placed on the AFM, ensuring they did not dry at any time. Force curves were then obtained while randomly varying acquisition parameters like the ramp length, the maximum applied load, the sampling frequency and the ramp velocity.

#### ML analysis of force curves

The contact point of all force curves was manually labelled with a custom made application available at https://t.ly/Y0hC.

Before feeding the force curves to the neural networks, they were interpolated to a final size of 5120 points, and subsequently normalized in the vertical and normal directions. The labelled contact point was also encoded by the normalized sample position at which it occurred,

The 1D CNNs architectures depicted in Fig. 1 were implemented using Keras^57^. The specific implementation is available in the repository of this work: https://t.ly/MkVf. For training, we used mse loss and Adam optimization with an initial learning rate of 10^–4^, a 1st momentum of 0.9 and a 2nd momentum of 0.999. All models were trained and validated on a system with the following characteristics: Intel Core i9 7980XE (18 cores, 36 threads) CPU, 64 GB of DDR4 RAM and a GPU NVIDIA 2080 RTX Ti 11 GB GDDR6 (4352 computation cores). Models were always trained and evaluated using the GPU.

## Data Availability

All used force curves and code can be found in the repository of this work, available at: https://t.ly/MkVf.
